# Evolutionary dynamic analyses on monocot flavonoid 3′-hydroxylase gene family reveal evidence of plant-environment interaction

**DOI:** 10.1186/s12870-019-1947-z

**Published:** 2019-08-08

**Authors:** Yong Jia, Bo Li, Yujuan Zhang, Xiaoqi Zhang, Yanhao Xu, Chengdao Li

**Affiliations:** 1 State Agricultural Biotechnology Centre (SABC), School of Veterinary and Life Sciences, Murdoch University, Murdoch, WA 6150 Australia; 2 Western Barley Genetic Alliance, Murdoch University, Murdoch, WA 6150 Australia; 3 Hubei Collaborative Innovation Centre for Grain Industry, Yangtze University, Jingzhou, 434025 Hubei China; 4 Department of Primary Industry and Regional Development, Government of Western Australia, South Perth, WA 6155 Australia

**Keywords:** Flavonoids, Flavonoid 3′-hydroxylase, Anthocyanin, Cereal crops, Evolutionary dynamics, Gene evolution, Positive natural selection, Plant environmental interactions

## Abstract

**Background:**

Flavonoid 3′-hydroxlase (F3’H) is an important enzyme in determining the B-ring hydroxylation pattern of flavonoids. In monocots, previous studies indicated the presence of two groups of F3’Hs with different enzyme activities. One F3’H in rice was found to display novel chrysoeriol-specific 5′-hydroxylase activity. However, the evolutionary history of monocot F3’Hs and the molecular basis for the observed catalytic difference remained elusive.

**Results:**

We performed genome-wide survey of 12 common monocot plants, and identified a total of 44 putative *F3’H* genes. The results showed that *F3’H* gene family had underwent volatile lineage-specific gene duplication and gene loss events in monocots. The expansion of *F3’H* gene family was mainly attributed to dispersed gene duplication. Phylogenetic analyses showed that monocot *F3’Hs* have evolved into two independent lineages (Class I and Class II) after gene duplication in the common ancestor of monocot plants. Evolutionary dynamics analyses had detected positive natural selection in Class II *F3’Hs*, acting on 7 specific amino acid sites. Protein modelling showed these selected sites were mainly located in the catalytic cavity of F3’H. Sequence alignment revealed that Class I and Class II F3’Hs displayed amino acid substitutions at two critical sites previously found to be responsible for F3’H and flavonoid 3′5’-hydroxylase (F3’5’H) activities. In addition, transcriptional divergence was also observed for Class I and Class II *F3’Hs* in four monocot species.

**Conclusions:**

We concluded that monocot *F3’Hs* have evolved into two independent lineages (Mono_F3’H Class I and Class II), after gene duplication during the common ancestor of monocot plants. The functional divergence of monocot F3’H Class II has been affected by positive natural selection, which acted on specific amino acid sites only. Critical amino acid sites have been identified to have high possibility to affect the substrate specificity of Class II F3’Hs. Our study provided an evolutionary and protein structural explanation to the previously observed chrysoeriol-specific 5′-hydroxylation activity for CYP75B4 in rice, which may also be true for other Class II F3’Hs in monocots. Our study presented clear evidence of plant-environmental interaction at the gene evolutionary level, and would guide future functional characterization of F3’Hs in cereal plants.

**Electronic supplementary material:**

The online version of this article (10.1186/s12870-019-1947-z) contains supplementary material, which is available to authorized users.

## Background

Flavonoids including anthocyanins, flavones and flavonols are ubiquitous secondary metabolites present in all organs and tissues of plants [1, 2]. During the past few decades, enormous research attention has been drawn toward their biological functions in monocot cereal crops [1, 3, 4], such as wheat, barley, rice and maize, which are the major sources of human food. From the biological perspective, flavonoid biosynthesis plays important roles in plant’s defence mechanism to various abiotic and biotic stress factors including UV-radiation, heat, heavy metal ions, drought, pathogen and microbial invasion et al. [5–7]. Flavonoid pigments in flower and seed are visible signals to attract insects and animals for pollination and seed dispersal [8, 9]. In addition, flavonoids have been shown to be involved in pollen germination [10, 11], and could also function as developmental regulators in auxin transport and catabolism [12, 13]. Flavonoids such as anthocyanin accumulation in cereal grains has been shown to affect seed dormancy and prevent preharvest sprouting [3, 14], which assists plant’s survival in unfavourable environmental conditions. From the food consumption perspective, flavonoid compounds, due to their antioxidant properties, also have demonstrated great health benefits in the protection of degenerative diseases such as coronary heart disease and cancer [15–17].

The molecular mechanisms of flavonoid biosynthesis has been well established in monocot plants [1]. As the starting point, phenylalanine was transformed via the phenylpropanoid pathway into 4-coumaroyl-CoA, which then enters the flavonoid biosynthesis pathway [18]. Chalcone synthase (CHS) and chalcone isomerase (CHI) are the first two enzymes in the flavonoid pathway, leading to the sequential production of chalcones and naringenin, which act as the precursors for all flavonoid classes [19]. Based on the hydroxylation pattern of the flavonoid B-ring, flavonoid biosynthesis can diverge into three different directions, resulting in the final production of one-hydroxy (pelargonidin-type), two-hydroxy (cyaniding-type) and three-hydroxy (delphinidin-type) anthocyanins [20]. The hydroxylation pattern of the flavonoid B-ring is controlled by two key enzymes flavonoid 3-hydroxylase (F3’H) and flavonoid 3′5’-hydroxylase (F3’5’H). F3’H belongs to the CYP75B subfamily in the cytochrome P45O-dependent monooxygenase superfamily, while F3’5’H belongs to the CYP75A subfamily and represents a lateral functional divergence from F3’H [21]. In the flavonoid pathway, F3’H catalyses the hydroxylation of naringenin and dihydrokampferol at the 3′-position, leading to the final production of cyanidin-based anthocyanins. Instead, F3’5’H is able to hydroxylate the flavonoid B-ring at both 3′ and 5′ position, which is responsible for the delphinidin-based anthocyanin production. The F3’H activity, together with the F3’5’H activity, compete with the central flavonoid pathway without hydroxylation, and have led to the great diversification of the flavonoid biosynthesis pathway [20]. This metabolic diversification has been suggested to play a critical role in plant’s adaption to the diverse environmental conditions during evolution.

Gene duplication is a widespread phenomenon in plant genomes. It generates the raw genetic material for environmental selection to act upon, playing a central role in plant diversification, thus facilitating their environmental adaptation [22, 23]. Following duplication, the gene duplicates could be either lost or retained, depending on whether beneficial function could arise or not at either the protein structural level and/or the gene transcriptional level. The retention of species-specific gene copy number has often been proposed to assist different plants to meet their specific environmental challenges. A recent study reported that 2 copies of *F3’Hs* (*F3’H-1* & *F3’H-2*) were present in barley genome, which were resulted from a duplication event before the divergence of *Triticeae* tribe [24]. A tissue-specific expression profile was also observed for *F3’H-1* and *F3’H-2*. In another earlier study, 3 and 2 copies of *F3’Hs* has been identified in barley and rice, respectively [25]. Interestingly, one of the two F3’Hs in rice (CYP75B4) was proven to have recruited novel 5′-hydroxlase activity on chrysoeriol, which comprised a critical step in tricin biosynthesis [25]. Preliminary phylogeny analysis showed CYP75B4 belonged to an independent phylogenetic group divergent from the normal monocot F3’Hs. CYP75B3 and CYP75B4 in rice were also shown to have different substrate specificity [26]. These observations suggested a potential functional divergence among monocot F3’Hs. However, no systematic and comprehensive evolution analyses have been performed on F3’H gene family in monocot. The protein structural basis underlying the 5′-hydroxylase activity of CYP75B4 remained to be characterised.

In this study, we investigated the conservation of putative F3’H genes in the major cereal plants, for which the genomic data is available in the public databases. The evolutionary history of F3’H genes in monocot plants was characterized by comprehensive phylogenetic and natural selection analyses. We found clear evidence of plant-environmental interaction during the evolution of monocot F3’H gene subfamily. Our study consolidated the flavonoid biosynthesis pathway as a model to investigate plant and environment interaction, and would also serve as a guide for future functional study on the F3’H gene subfamily in monocot plants.

## Results

### Identification of F3’H in monocot plants

To identify the genuine F3’H genes, a comprehensive Neighbour Joining tree was developed based on the amino acid sequence alignment of the retrieved F3’H homologs. Two distinct branches encompassing the previously characterised F3’Hs and F3’5’Hs, respectively, were identified. Those homologous proteins in the F3’H branch were considered genuine *F3’Hs* and were selected for further analyses. As summarised in Table 1, a total of 44 putative *F3’H* genes were identified from 12 monocots. At least two copies of *F3’H* were present in each species. The highest number of *F3’H* occurred in *Triticum aestivum* (9), followed by *Triticum dicoccoides* (7) in the *Triticeae* subfamily. All the other *Triticeae* crops including *Hordeum vulgare*, *Aegilops tauschii* and *Secale cereale* contain 3 copies of F3’H genes, with the exception of *Triticum urartu*, which has 2 copies instead. *Brachypodium distachyon*, a close relative to *Triticeae*, contained 4 copies of *F3’Hs*. In addition, most plants in the *Panicoideae* lineage, including *Setaria italica* and *Panicum hallii*, retain 2 *F3’Hs*, whilst *Zea mays* and *Sorhum bicolor* have exceptionally 3 and 5 copies, respectively. Notably, the other important crop *Oryta sativa* also contained 2 *F3’Hs*.

**Table 1:** Identification of putative F3’H genes in cereal plants. The F3’H class was classified based on the phylogeny analyses. The gene duplication pattern was determined using the MCScanX tool. NA stands for “not applicable”

Species		Chr	F3’H gene ID	F3’H class	Duplication pattern	Start position	End position
*Triticeae*	*H. vulgare*	1H	HORVU1Hr1G094880	Class I	Dispersed duplication	556,691,949	556,693,904
		6H	HORVU6Hr1G002400	Class II	Dispersed duplication	6,328,532	6,330,799
		7H	HORVU7Hr1G095900.47	Class II	Dispersed duplication	585,061,615	585,071,468
	*A. tauschiii*	Aet1	AET1Gv21041800	Class I	Dispersed duplication	499,122,364	499,126,756
		Aet6	AET6Gv20027200	Class II	Dispersed duplication	5,657,246	5,659,238
		Aet7	AET7Gv20999800	Class II	Dispersed duplication	523,485,446	523,487,617
	*T. turgidum*	Tt1A	TRIDC1AG064860	Class I	NA	590,437,663	590,440,408
		Tt1B	TRIDC1BG074020	Class I		685,939,174	685,944,027
		Tt2B	TRIDC2BG088640	Class I		792,785,227	792,787,184
		Tt6A	TRIDC6AG001340	Class II		4,624,395	4,644,086
		Tt6B	TRIDC6BG002010	Class II		11,399,578	11,402,254
		Tt7A	TRIDC7AG057400	Class II		599,327,799	599,328,465
		Tt7B	TRIDC7BG049820	Class II		562,791,603	562,792,604
	*T. aestivum*	Ta1A	TraesCS1A01G442300.1	Class I	NA	590,995,642	590,997,413
		Ta1B	TraesCS1B01G476400.1	Class I		685,231,562	685,233,491
		Ta1D	TraesCS1D01G450100.1	Class I		492,534,241	492,538,011
		Ta2B	TraesCS2B01G613200.1	Class I		792,677,476	792,679,409
		Ta6A	TraesCS6A01G012600.1	Class II		5,861,572	5,863,417
		Ta6B	TraesCS6B01G018800.1	Class I		11,574,703	11,578,097
		Ta6D	TraesCS6D01G015200.1	Class II		6,319,419	6,321,226
		Ta7A	TraesCS7A01G411700.1	Class II		602,804,667	602,806,415
		Ta7D	TraesCS7D01G404900.1	Class II		522,502,518	522,504,208
	*S. cereale*	Sc1	Sc1Loc01465431	Class I	NA	Lo7_v2_contig_2871825	
		Sc2	Sc2Loc01684522	Class I		Lo7_v2_contig_326626	
		Sc7	Sc7Loc01952123	Class II		Lo7_v2_contig_61986	
	*T. urartu*	Tu1	TuG1812G0100004862	Class I	Dispersed duplication	581,402,997	581,404,997
		Tu7	TuG1812G0700004460	Class II	Dispersed duplication	590,301,744	590,303,739
	*B. distachyon*	Bd1	Bradi1g17180	Class I	Dispersed duplication	13,787,434	13,789,806
		Bd1	Bradi1g24840	Class II	Dispersed duplication	20,108,563	20,112,348
		Bd3	Bradi3g04750	Class I	Dispersed duplication	3,260,666	3,262,706
		Bd4	Bradi4g16560	Class II	Dispersed duplication	17,368,956	17,372,786
	*O. sativa indica*	Os10	LOC_Os10g17260	Class I	Proximal duplication	8,679,309	8,681,284
		Os10	LOC_Os10g16974	Class II	Proximal duplication	8,494,247	8,504,329
*Panicoideae*	*Z. mays*	Zm4	Zm00008a016611	Class I	WGD/Segmental	131,908,959	131,910,431
		Zm5	Zm00008a022212	Class I	WGD/Segmental	177,289,973	177,292,210
		Zm8	Zm00008a031477	Class II	Dispersed duplication	116,235,840	116,239,418
	*S. bicolor*	Sb4	Sobic.004G200800	Class I	Tandem duplication	55,221,098	55,224,686
		Sb4	Sobic.004G200833	Class I	Tandem duplication	55,225,513	55,227,179
		Sb4	Sobic.004G200900	Class I	Proximal duplication	55,233,582	55,236,702
		Sb4	Sobic.004G201100	Class I	Proximal duplication	55,261,682	55,264,545
		Sb9	Sobic.009G162500	Class II	Dispersed duplication	51,943,204	51,948,939
	*S. italica*	Si9	Seita.9G244600	Class II	Dispersed duplication	19,091,837	19,094,929
		Si9	Seita.9G242900	Class I	Dispersed duplication	18,990,913	18,992,801
	*P. hallii*	Ph8	Pahal.H01052	Class II	Dispersed duplication	31,682,928	31,687,178
		Ph9	Pahal.I03232	Class I	Dispersed duplication	16,748,267	16,750,212

### Phylogeny inference

**Fig. 1:**
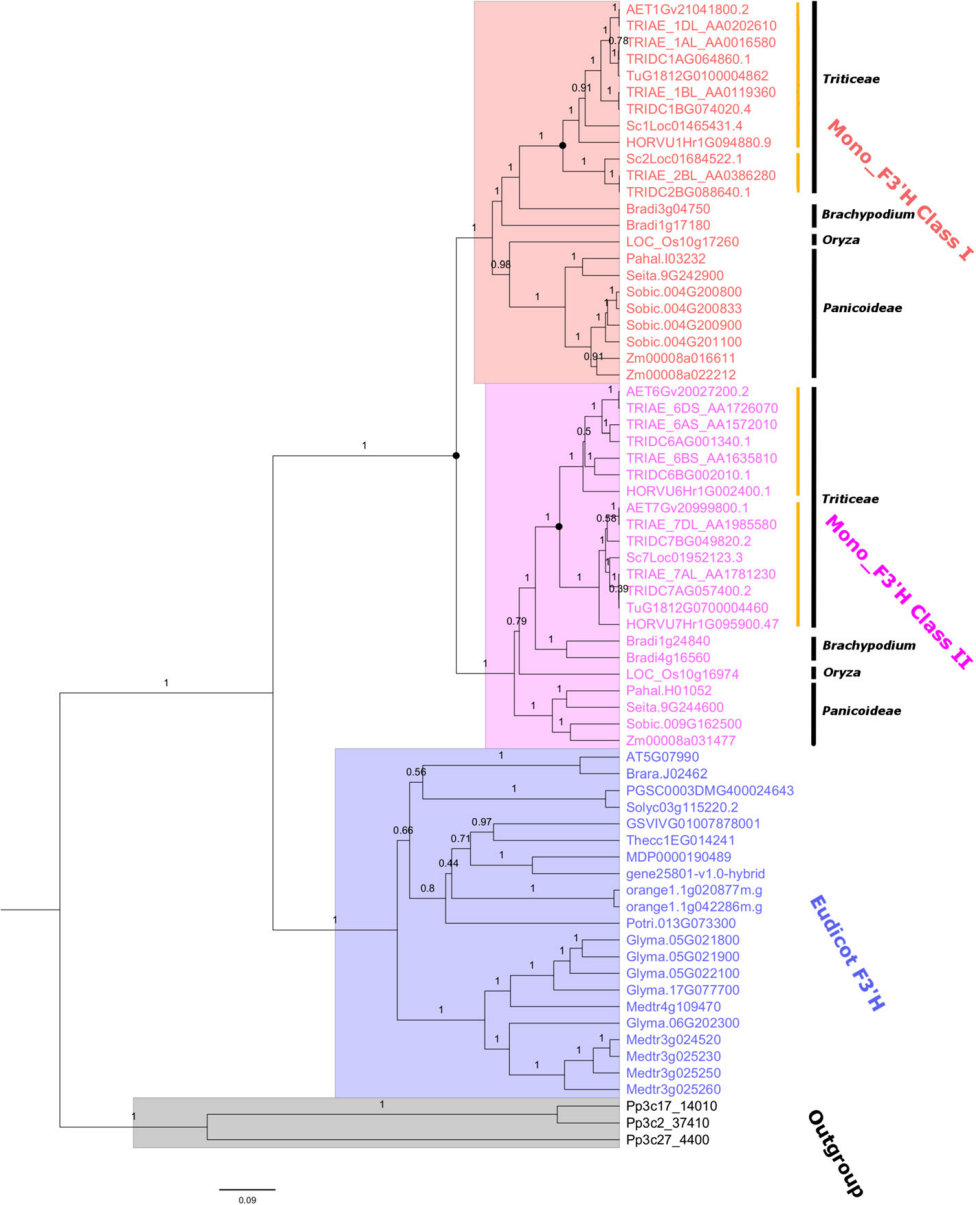
Phylogenetic tree displaying the evolutionary history of plant F3’Hs. The phylogeny was developed using Bayesian method. F3’H homologs from *P. patens* was included as outgroup. Posterior support was displayed above each branch. The deduced duplication event was indicated by solid black dot symbol. Yellow vertical lines indicated the division of sub-branches of *F3’Hs* in *Triticeae*

To investigate the evolutionary history of the F3’H gene family in monocot plants, a Bayesian phylogeny was developed based on the coding domain sequence (CDS) alignment of the identified *F3’Hs*. Eudicot *F3’Hs* and the remote *F3’H* homologs from the lower plant *Physcomitrella patens* were included as the out-group. Overall, the phylogenetic tree demonstrated a strong topology support, indicating the resolved phylogeny was highly reliable. As shown in Fig. 1, the target *F3’Hs* were grouped into two major clusters, corresponding to eudicot *F3’H* and monocot *F3’H*, respectively. The monocot *F3’Hs* further separated into two distinct lineages, which were classified here as Mono_F3’H Class I and Class II, respectively. Noteworthy, each of Mono_F3’H Class I and Class II covered all the monocot species included in the present study, suggesting the divergence occurred before the species diversification. A closer inspection on the phylogeny showed that these two lineages shared the same evolutionary pattern that resembles the species phylogeny of monocot plants. Specifically, within both Mono_F3’H Class I and Class II, *Panicoideae* plants including *Z. mays*, *S. bicolor* and *S. italica* diverged firstly, followed by *O. sativa*, which represents an evolutionary intermediate between *Panicoideae* and *Triticeae*. For the other plants, *B. distachyon* diverged before *Triticeae*. *F3’Hs* retrieved from *Triticeae* plants were clustered together with strong support. These results indicated that the Class I and Class II *F3’Hs* have evolved vertically within monocot plants, providing further support that the divergence between Class I and Class II *F3’Hs* have occurred during the common ancestor of monocot crops. The universal conservation of Class I and Class II *F3’Hs* among monocots indicated that both *F3’H* classes are essential for the normal growth of these plants.

The evolution of *F3’Hs* in monocots displayed a clear Class- and species-specific profile. Whilst only a single copy of both Class I and Class II *F3’Hs* were conserved within *Panicoideae* plants *S. italic* and *P. hallii*, *S. bicolor* and *Z. mays* contained 4 and 2 copies of Class I *F3’Hs,* respectively. In addition, lineage-specific expansion of *F3’H* were also observed in *B. distachyon*, which has 2 copies of both Class I and Class II *F3’Hs*. *O. sativa* resembled *S. italic* and *P. hallii* with one Class I and one Class II *F3’H*. For *Triticeae*, the evolution of *F3’H* seems to be independent from the above plants. Within the Mono_F3’H Class II lineage, *Triticeae* F3’Hs could be further divided into two sub-branches (yellow line; Fig. 1), which covered all the *Triticeae* plants included in this study. This indicated that Class II F3’Hs have underwent an extra round of duplication during the common ancestor of *Triticeae*, but after the divergence of *B. distachyon*. Noteworthy, a similar expansion pattern could be observed for *Triticieae* Class I *F3’Hs*, which had also evolved into two distinct sub-branches (yellow line; Fig. 1), indicating a duplication event predating the *Triticeae* diversification as well. One of the two sub-branches of Class I *F3’H* (the upper yellow line) covered all *Triticeae* plants, whilst the other is preserved only in *T. turgidum*, *T. aestivum* and *S. cereale*, but absent in *H. vulgare*, *A. tauschii*. The absence of the secondary sub-branch might be due to gene loss after duplication. Noteworthy, the identified duplication events for Class I and Class II F3’Hs in *Triticeae* may point to a shared genome-wide duplication event in the common ancestor of *Triticeae*. Further investigation is needed to verify this hypothesis. In addition to *Triticeae*, Class I and Class II *F3’Hs* in *B. distachyon* tended to have evolved independently as separate lineages.

### Genomic structure analyses

**Fig. 2:**
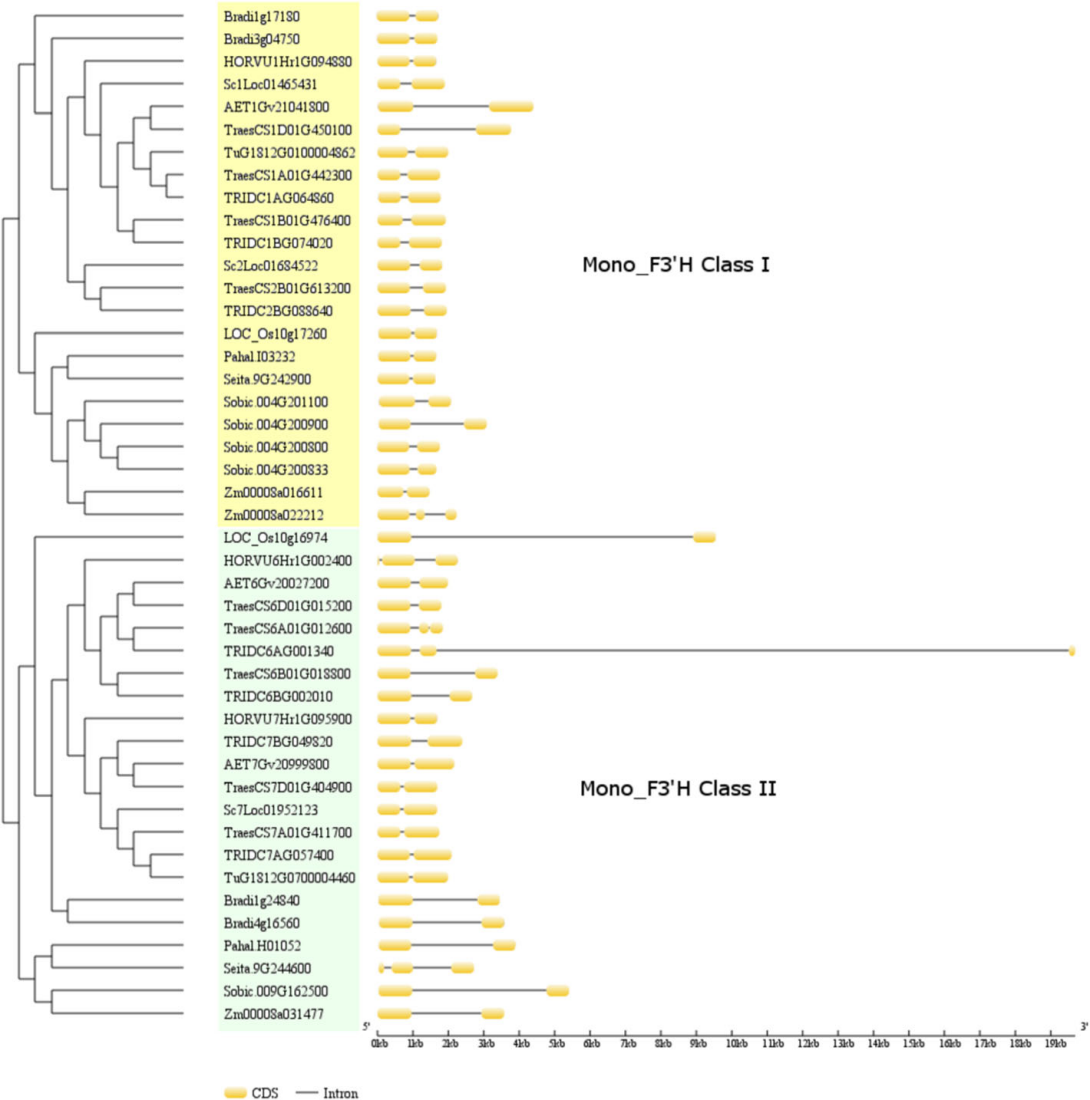
Genomic structures of monocot *F3’Hs*. The *F3’H* genes were clustered based on the developed phylogeny in the present study. Two features: CDS and Intron were displayed for each *F3’H* based data extracted from public database

To characterise the gene structural profiles of *F3’H* and their potential relation with the evolution history of monocot F3’H gene subfamily, the gene structures of monocot *F3’Hs* were analysed based on the developed phylogeny. As shown in Fig. 2, majority (41/45) of monocot *F3’Hs* contained two exons, regardless of the phylogeny groups. The other four putative *F3’Hs*, corresponding to Zm00008a022212 in Class I and TraesCS6A01G012600, TRIDC6AG001340, Seita.9G244600 in Class II, retained three exons. The intron length tends to be conserved for most *F3’Hs* from different phylogeny branches, with the exception of non-*Triticeae* Class II *F3’Hs*, which displayed a clear and universal increase in intron length. In addition to the non-*Triticeae* Class II *F3’Hs*, several other *F3’Hs* from both Class I and Class II also showed an increase in intron length, which corresponded to AET1Gv21041800, TraesCS1D01G450100, Sobic.004G200900 (Class I) and TraesCS6B01G018800, TRIDC6BG002010 (Class II). Of these five genes, AET1Gv21041800/TraesCS1D01G450100 and TRIDC6BG002010/TraesCS6B01G018800 are close homolog pairs and may have reflected the origin of *T. aestivum* D and B subgenomes from *A. tauschii* and *T. turgidum*, respectively. It should be noted that the gene structure data presented here is based on the gene annotation in the plant genome databases. Laboratory gene cloning and sequencing in respective species are needed to further validate these results. We also refrained to compare the putative promoter regions including the 5’UTR and 3’UTR of *F3’Hs* due to the lack of experimental information.

### Duplication pattern and synteny analyses

Gene duplicates arising from different mechanisms can be divided into four categories: Whole genome duplication (WGD)/segmental duplication, tandem duplication, proximal duplication and dispersed duplication. To further investigate the evolutionary history of *F3’H* family, gene duplication pattern were determined for *F3’H*s in 9 monocot species (Table 1). *T. aestivum*, *T. turgidum* and *S. cereale* were excluded for this analysis due to either their multi-ploidity or the lack of fine genome annotation information. As shown in Table 1, all of the *F3’Hs* in *Triticeae* plants were identified as dispersed duplicates. The same observation was made with the *F3’Hs* in *B. distacyon* (4 copies), all of which had arisen from dispersed gene duplication. *F3’Hs* in rice (2 copies), located close to each other on chromosome 10, were found to be proximal duplicates. Interestingly, 2 *F3’Hs* (Zm00008a016611, Zm00008a022212) from *Z. mays* were found to have originated from whole-genome or segmental duplication, reflecting a different evolution origin for F3’Hs in this species. The other *F3’H* (Zm00008a031477) in *Z. mays* was identified as a dispersed duplicate as well. In addition, 4 *F3’Hs* on chromosome 4 in *S. bicolor* were identified as tandem duplication or proximal duplication, which was quite unusual compared to other species. The other *F3’H* (Sobic.009G162500) in *S. bicolor* was found as a dispersed duplicate. Unlike *Z. mays* and *S. bicolor*, all of the *F3’Hs* from *S. italic* and *P. hallii* in the *Panicoideae* tribe were found to have resulted from dispersed duplication.

**Fig. 3:**
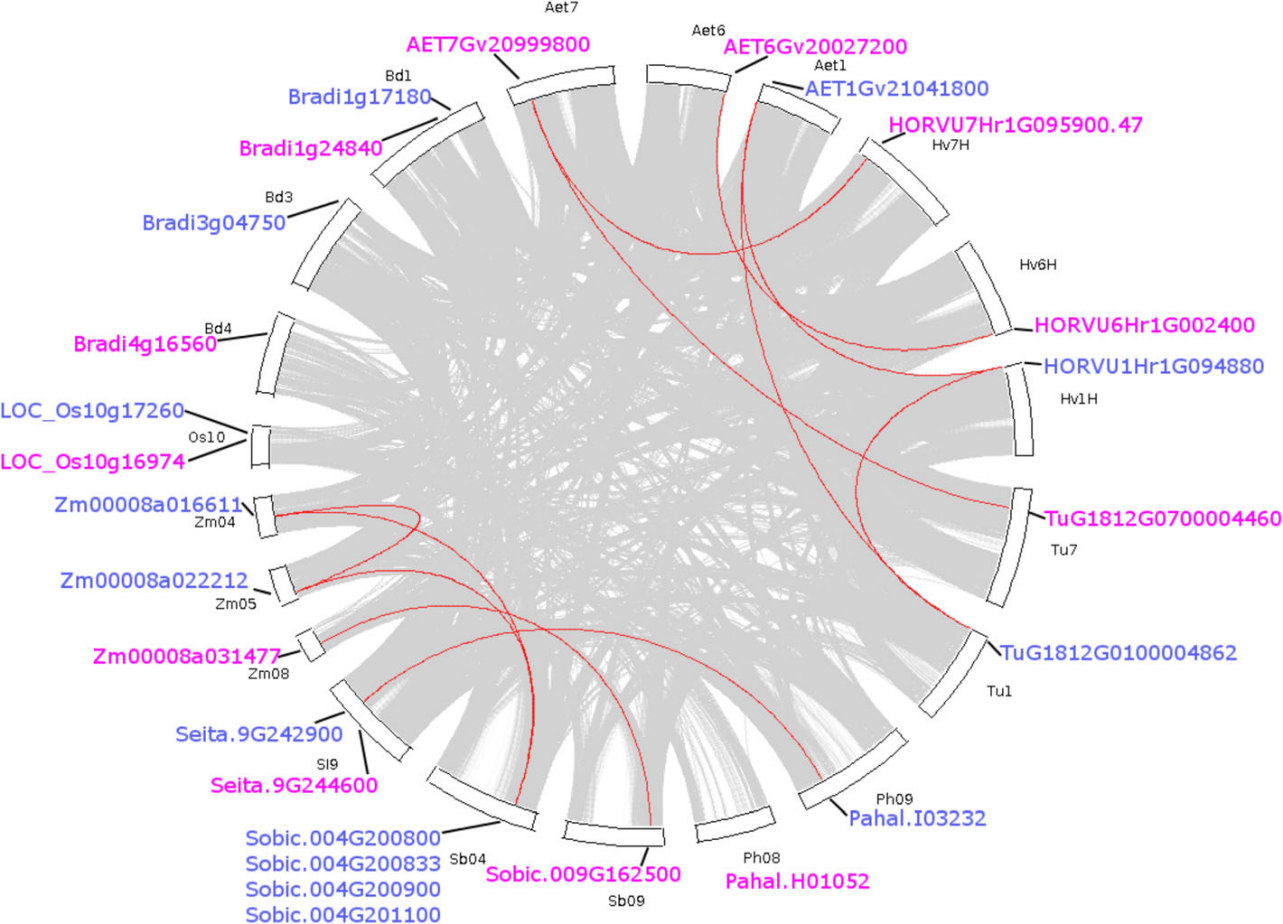
Displays the collinear *F3’H* gene pairs across monocot plants. The circle plot was created by MCScanX tool. Identified colinear genes were linked by red curved lines. Hv, Bd, Aet, Tu, Os, Zm, SI, Sb and Ph represent *H. vulgare*, *B. distachyon*, *A. tauschii*, *T. urartu*, *O. sativa*, *Z. mays*, *S. italica*, *S. bicolor* and *P. hallii*, respectively. Only the chromosomes containing *F3’H* genes were included for this analysis. Class I and Class II F3’Hs were labelled in blue and pink colours, respectively

To investigate the syntenic conservation of *F3’Hs* across monocot plants, collinear *F3’H* gene pairs were identified. As shown in Fig. 3, a total of 12 collinear *F3’H* pairs have been identified for *F3’Hs* in 9 monocot species. These gene pairs could be divided into two clusters: *Triticeae*-specific and *Panicoideae*-specific. For *Triticeae*, *F3’Hs* located on chromosome Hv1H, Hv6H and Hv7H in *H. vulgare* were found to be collinear with *F3’Hs* on chromosome Aet1, Aet6 and Aet7 in *A. tauschii*, respectively. Noteworthy, TuG1812G0100004862 on chromosome Tu1 in *T. urartu* was located in a collinear region with HORVU1Hr1G094880 and AET1Gv21041800, TuG1812G0100004862 on chromosome Tu7 was collinear with AET7Gv20999800, which also formed collinear pair with HORVU7Hr1G095900.47. Interestingly, all these gene pairs occurred within the same class of *F3’Hs*. No inter-class *F3’H* pair had been observed in *Triticeae*. Taken together, the identified *F3’H* collinear pairs reflected the close relationship among *Triticeae* species and also demonstrated the strict conservation of *F3’Hs* in these plants. Noteworthy, no F3’H has been found on chromosome Tu6 in *T. urartu*, which may have been lost in this species.

The other identified collinear F3’H pairs were found in *Panicoideae* only (Fig. 3). No collinearity could be identified for the F3’Hs in *O. sativa* and *B. distacyon,* which may reflect a divergent genomic location for the F3’Hs in these two species. For *Panicoideae* including *Z. mays, S. italic, S. bicolor* and *P. hallii*, collinearity was mainly found for Class II *F3’Hs*, with one intro-species pair (Zm00008a016611-Zm00008a022212) identified in *Z. mays*. In addition, only one collinear pair (Zm00008a031477-Sobic.009G162500) was found for Class I *F3’Hs* in *Panicoideae*. No collinearity could be observed for the Class I *F3’Hs* in *S. italica* (Seita.9G244600) and *P. hallii* (Pahal.H01052). Again, no inter-class collinearity could be identified between Class I and Class II *F3’Hs* in *Panicoideae*.

### Natural selection test

To investigate the evolutionary dynamics in monocot *F3’H* gene family, natural selection tests were performed on the developed *F3’H* phylogeny. The ratio (ω) of non-synonymous and synonymous substitution is an important parameter to assess the selection pressure on evolving genes, whereby ω < 1, ω = 1 and ω > 1 indicate purifying selection, neutral evolution and positive selection, respectively. Branch and amino acid site specific ω values were calculated for Monocot F3’H Class I and Class II under different hypotheses. Eudicot *F3’H* was used as the reference. For the branch specific models, three hypotheses (Table 2) were tested. Likelihood-Ratio Tests (LRTs) showed that the two-ratio models ω_[eudi]_ = ω_[mono1]_ ≠ ω_[mono2]_ and ω_[eudi]_ = ω_[mono2]_ ≠ ω_[mono1]_, which specified divergent ω values for Monocot F3’H Class I and Class II, respectively, were both significantly better (df = 1, *p* < 0.0001; df = 1, *p* = 0.0444) than the one ratio model ω_[eudi]_ = ω_[mono1]_ = ω_[mono2]_. In addition, the three ratio model ω_[eudi]_ ≠ ω_[mono1]_ ≠ ω_[mono2]_, specifying different ω values for all the three branches, fit the dataset significantly (df = 1, *p* < 0.0001) better than ω_[eudi]_ = ω_[mono2]_ ≠ ω_[mono1]_, but not better (df = 1, *p* = 1.0) than ω_[eudi]_ = ω_[mono1]_ ≠ ω_[mono2]_. These calculations indicated that ω_[mono2]_ was significantly different from ω_[eudi]_ and ω_[mono1]_, whilst ω[eudi] and ω[mono2] were not significantly different from one another. This suggested that Monocot Class II *F3’Hs* had underwent significantly different selection pressure compared to Monocot Class II *F3’Hs* and Eudicot *F3’Hs*. Under the best-fitting model ω_[eudi]_ = ω_[mono1]_ ≠ ω_[mono2]_, ω_[mono2]_ was calculated as 0.82808, while ω_[eudi]_ and ω_[mono1]_ equalled 0.11857. These calculations indicated that Monocot Class I F3’Hs and Eudicot F3’Hs were under strong purifying selection, whilst Monocot Class II F3’Hs were relatively more divergent.

**Table 2:** Natural selection tests on plant F3’Hs. “np” stands for the number of parameters. *ln*(Likelihood) refers the log value of the likelihood

Model	np	*ln*(Likelihood)	Estimates of parameters^a^ (ω = d_N_: d_S_; P – percentage of site)	Positively selected sites^b^
One-ratio				
ω_[eudi]_ = ω_[mono1]_ = ω_[mono2]_	1	−29,872.87	ω_[eudi]_ = ω_[mono1]_ = ω_[mono2]_ = 0.12094	Not Allowed (NA)
Branch-specific models				
ω_[eudi]_ = ω_[mono1]_ ≠ ω_[mono2]_	2	−29,862.42	ω_[eudi]_ = ω_[mono1]_ = 0.11857, ω_[mono2]_ = 0.82808	NA
ω_[eudi]_ = ω_[mono2]_ ≠ ω_[mono1]_	2	−29,870.85	ω_[eudi]_ = ω_[mono2]_ = 0.11990, ω_[mono1]_ = 0.43835	NA
ω_[eudi]_ ≠ ω_[mono1]_ ≠ ω_[mono2]_	3	− 29,862.42	ω_[eudi]_ = 0.11853, ω_[mono2]_ = 0.78501, ω_[mono1]_ = 0.12479	NA
Site-specific models				
Neutral M1 (2 site classes)	2	−29,413.91	P_0_ = 0.87805 (P_1_ = 1-P_0_ = 0.12195); ω_0_ = 0.09334 (ω_1_ = 1- ω_0_ = 1.0)	NA
Selection M1 (3 site classes	3	−29,413.91	P_0_ = 0.87805, P_1_ = 0.01421, P_2_ = 1-P_0_-P_1_ = 0.10774; ω_0_ = 0.09334 (ω_1_ = 1.0), ω_2_ = 1.0	NA
Branch-site models				
Model A Null (Class I F3’H)	3	−29,408.41	P_0_ = 0, P_1_ = 0.0, P_2_ + P_3_ = 1; ω_0_ = 0.09152, ω_1_ = 1.0, ω_2_ = 1.0	
Model A (Class I F3’H)	4	−29,408.41	P_0_ = 0.00005, P_1_ = 0.00001, P_2_ + P_3_ = 0.99994; ω_0_ = 0.09152, ω_1_ = 1.0, ω_2_ = 1.0	None
Model A Null (Class II F3’H)	3	−29,394.83	P_0_ = 0.51238, P_1_ = 0.07209, P_2_ + P_3_ = 0.41553; ω_0_ = 0.09046, ω_1_ = 1.0, ω_2_ = 1.0	NA
Model A (Class II F3’H)	4	−29,392.04	P_0_ = 0.68799, P_1_ = 0.09608, P_2_ + P_3_ = 0.21593; ω_0_ = 0.09035, ω_1_ = 1.0, ω_2_ = 8.19737	108R,222A, 265 V, 274 T, 355Q, 447S, 449 L (*p* < 0.05)

^a^In the site-specific model M1, two site classes were specified: highly conserved sites (ω_0_) and neutral sites (ω_1_ = 1). For the site-specific model M2, there were three site classes: highly conserved sites (ω_0_), neutral sites (ω_1_ = 1) and positively selected sites (ω_2_). In Model A, four site classes were specified. The first two classes had ω ratios of ω_0_ and ω_1_ respectively, corresponding to highly conserved sites and neutral sites across all lineages. In the other two site classes, the background lineages had ω_0_ or ω_1_ while the foreground lineages had ω_2_. ^b^Positively selected amino acids at *P*-value ≤0.05 are numbered according to HORVU6Hr1G002400.1, excluding the first 34 amino acids predicted as membrane targeting signal

To further characterise the evolutionary dynamics of monocot F3’Hs, the site-specific model, which allows the ω value to vary along different amino acid sites, were applied to the same dataset. Results (Table 2) showed that Selection M1 was not better (df = 1, *p* = 1.0) than the neutral M1. No amino acid site could be identified to be under positive selection in the selection model. To test whether ω may vary at specific amino acid sites in specific branches, branch-site models were also tested (Table 2). When Monocot Class II *F3’H* was set as the foreground branch, the selection Model A revealed that 7 amino acid sites (108R, 222A, 265 V, 274 T, 355Q, 447S, 449 L) in the Monocot Class II *F3’H* branch were under positive selection (ω _2_ = 8.19737; *p* < 0.05). LRTs showed that Model A was significantly better than its null hypothesis Model A Null, which specified ω _2_ = 1.0. Comparison of Model A with Neutral M1 (df = 2, *p* < 0.0001) also supported that these amino acid sites were under positive selection. In contrast, when Monocot Class I *F3’H* was set as the foreground branch, no amino acid site could be identified to be under positive selection at the significant level (Table 1). In this case, the selection Model A did not fit the dataset better (df = 1, *p* = 1.0) than its null hypothesis Model A Null. Taken together, natural selection assessments indicated that Monocot Class II F3’Hs were under significantly positive selection, which had been detected only in Monocot Class II F3’Hs, affecting specific amino acid sites in this branch.

### Sequence alignment and protein modelling analyses

**Fig. 4:**
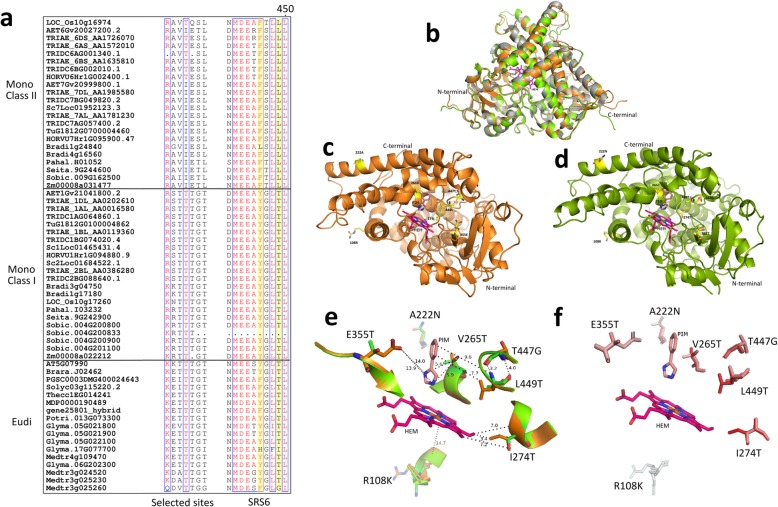
Sequence alignment and protein modelling analyses. **a** Amino acid sequence alignment of the selected sites and SRS6 in plant F3’Hs. **b** The overall superimposition of the LOC_Os10g16974 (orange) and LOC_Os10g17260 (green) models with CYP76AH1 (grey; PDB: 5YM3); **c** The spatial location of the positively selected sites in LOC_Os10g16974; **d** The spatial location of the positively selected sites in LOC_Os10g17260; **e** The potential interaction of the positively selected sites with the superimposed substrates; **f** The hydrophobicity changes between LOC_Os10g16974 and LOC_Os10g17260 at the positively selected amino acid residues. Red and white colours represent the most hydrophobic and the most hydrophilic, respectively. HEM stands for heme. PIN refers enzyme inhibitor in PDB 5YM3

The amino acid substitutions at the positively selected sites between Class I and Class II F3’Hs were analysed by sequence alignment. As shown in Fig. 4a, monocot Class I and Class II F3’Hs displayed clear amino acid substitutions at 6 of the 7 selected sites. For all of these sites, Class I F3’Hs resembled eudicot F3’Hs, which differed from Class II F3’Hs, suggesting a closer relationship between Class I F3’Hs and eudicot F3’Hs. The enzyme activity of F3’Hs could be affected by critical amino acid sites. Previous studies have identified 6 substrate recognition sites (SRS1-SSR6) for Cytochrome P450s proteins. Sequence alignment showed that 4 of the 7 selected amino acid sites were located in SRS4 (265 V, 274 T) and SRS6 (447S, 449 L), suggesting direct potential to affect the enzyme activity of Class II F3’Hs. Moreover, two amino acid sites (yellow highlight in Fig. 4a) in SRS6 have been shown to be critical to determine the activity of F3’H and F3’5’H. As shown in Fig. 4a, most Class II F3’Hs contained 446F-449L, whilst Class I F3’Hs displayed 446Y-449 T. Interestingly, the T446F substitution has previously been shown to be able to enable 5′-hydroxylation activity in some eudicot F3’Hs [27]. The conservation of Thr at position 449 was also considered critical for the 3′-hydroxylation activity in F3’Hs [27]. It should also be noted that the amino acids at position 447 and 449 were identified to be affected by positive natural selection in Class II F3’Hs. Taken together, these observations indicated a strong potential of functional divergence for Class II F3’Hs. In addition, SRS6 was found to be missing in Sobic.004G200833 from *S. bicolor*, indicating that this Class I F3’H may not be functional.

To further investigate the potential effects of natural selection on the enzyme function of Class II F3’Hs, 3D structural models of the Class II (LOC_Os10g16974) and Class I (LOC_Os10g17260) F3’Hs in rice were developed by homology modelling. A recently determined CYP76AH1 crystal structure (PDB: 5YM3) was identified as a close homolog (~ 35% amino acid identity) to F3’H and was used as the template. The rice homologs were chosen due to the strict conservation of a single copy F3’H for each class in this species. As shown in Fig. 4b, the majority of the full length rice F3’Hs could be reliably modelled, with the exception of the short fragments at the N and C terminals. The un-modelled N terminal peptides were predicted as hydrophobic membrane binding domain and do not have direct effect on the enzyme function. The spatial location of the 7 amino acid sites were analysed, as displayed in Fig. 4c & d. Five out of the 7 amino acid sites were found to be located in the catalytic region (Fig. 4e), forming part of the catalytic cavity. The other two amino acid sites belonged to the N terminal region and were located on the exterior surface. Specifically, the side-chains of the 5 amino acid sites in the catalytic region were positioned toward the bound substrates heme and PIM with close distance (3.2 Å ~ 14.0 Å) and may have direct effect on the cavity volume and substrate binding. Structural superimposition revealed amino acid substitutions between LOC_Os10g16974 (Class I) and LOC_Os10g17260 (Class II) F3’Hs at all of the 7 amino acid sites. Noteworthy, four amino acid sites in the catalytic region of LOC_Os10g17260 were found to have the Threonine residue (neutral side chain), which were replaced with Valine (hydrophobic), Isoleucine (hydrophobic), Glutamic acid (charged side chain) and Leucine (hydrophobic). The hydrophobicity profiles of the selected sites were displayed in Fig. 4f. The amino acid substitutions V265 T, I274T, T447G and L449 T not only caused clear hydrophobicity changes between LOC_Os10g16974 and LOC_Os10g17260, but also affect the size of the catalytic cavity due to differences in the side chains. These observations strongly indicated a potential functional divergence after the split of the two classes of F3’Hs in monocot plants, which has been driven by positive natural selection.

In addition, based on the CYP76AH1 structure, the catalytic sites of monocot F3’Hs were identified by selecting the amino acid residues within a close distance (< 5 Å) to the bound heme molecule and enzyme inhibitor PIM. As a result, a total of 39 amino acid sites were identified (Additional file 1). Sequence alignment showed that, the majority of these putative catalytic sites were strictly conserved among all plant F3’Hs. However, an extra amino acid substitution between Class I and Class II F3’Hs was identified at position 84, which was proximal to the enzyme substrate.

### Transcriptional analyses

**Fig. 5:**
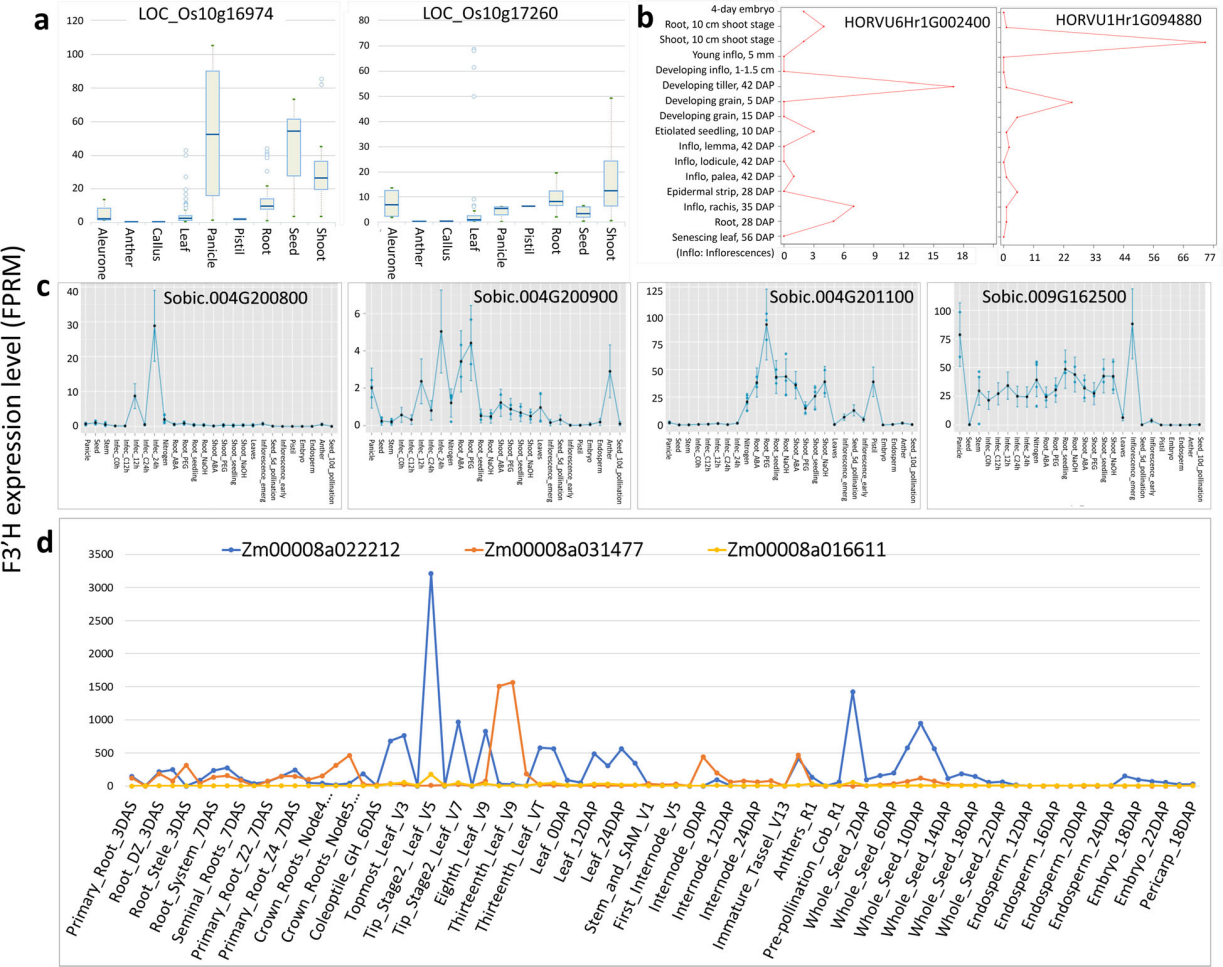
Transcriptional profiles of monocot *F3’Hs*. **a** LOC_Os10g16974 (Class II) and LOC_Os10g17260 (Class I) in rice; **b** HORVU6Hr1G002400 (Class II) and HORVU1Hr1G094880 (Class I) in barley; **c** Sobic.004G200800, Sobic.004G200833, Sobic.004G200900, Sobic.004G201100 (Class I) and Sobic.009G162500 (Class II) in Sorghum; **d** Zm00008a016611, Zm00008a022212 (Class I) and Zm00008a031477 (Class II) in maize

To explore the potential transcriptional divergence between Class I and Class II *F3’Hs*, the transcriptional data for monocot F3’Hs were searched in public databases. In rice (Fig. 5a), the Class I F3’H LOC_Os10g16974 was highly expressed in panicle, seed and shoot, relatively lower in root, and was barely expressed in other tissues such as anther, callus, leaf and pistil. Compared to LOC_Os10g16974, the Class II F3’H LOC_Os10g17260 generally displayed much lower expression in all tissues, except the pistil tissue, in which LOC_Os10g17260 had relatively higher transcription. The highest expression of LOC_Os10g17260 was found in shoot and root. It should be noted that, while the median expression level of LOC_Os10g17260 was very low in leaf, its expression could reach exceptionally high in some conditions. In barley, a clear transcriptional divergence for Class I and Class II F3’Hs was also observed (Fig. 5b). The Class I F3’H HORVU6Hr1G002400 was found to be mainly expressed in the developing tillers. Slight expression of HORVU6Hr1G002400 was also observed in inflorescences rachis and seedling root. In contrast, the Class II F3’H HORVU1Hr1G094880 was predominantly expressed in seedling shoots, followed by the developing grain at the early stage. Little expression of HORVU1Hr1G094880 was found in other tissues. An interesting observation with F3’Hs in sorghum (Fig. 5c) is that, the sorghum Class I F3’H Sobic.009G162500 was barely transcribed in all the reproductive tissues including anther, seeds, endosperm, embryo, pistil and the early inflorescence, with the exception of the emerging inflorescence. Instead, abundant expression of Sobic.009G162500 was found in the vegetative tissues panicle, stem, root and shoot. In contrast, Class II F3’Hs Sobic.004G201100 displayed moderate expression in pistil and young seeds. Significant transcription of Sobic.004G201100 was also found in vegetative tissues root and shoot. The other two Class II F3’Hs Sobic.004G200800 and Sobic.004G200900, which are tandem duplicates, had very low or barely no expression in all tissues studied. Noteworthy, although Sobic.004G200800 was barely expressed in sorghum, a dramatic increase of its expression was observed in leaves upon pathogen infection. In maize, the Class I and Class II F3’Hs also displayed a clear transcriptional divergence (Fig. 5d). The highest expression of Zm00008a031477 (Class I *F3’H*) was found in leaves, followed by moderate expression in root, internode and meiotic tassel. In contrast, Zm00008a022212 (Class II *F3’H*) displayed more widespread expression abundant in leave tips, silks and whole seeds at 10 days after pollination (DAP), and moderate in pooled leaf, topmost leaf and mature leave. Interestingly, the other Class II *F3’H* Zm00008a016611 was barely expressed in all tissues studied.

## Discussions

The evolution of complex metabolic pathways such as flavonoid biosynthesis has been indicated to play a critical role in plant evolution, helping plants adapt to various biotic and abiotic stressors [28, 29]. The flavonoid biosynthetic pathway has been extensively studied in di-cotyledon plants, whilst only moderate attention has been paid toward monocot cereal crops [1]. This observation is staggering, considering the fact that cereal plants such as wheat, rice, barley and maize comprise the most economically important food and feed sources for human and animals. Due to the ubiquitous presence of flavonoids in plant tissues and flavonoids being potent antioxidant, a close relationship between flavonoid biosynthesis and environmental adaptation has also been established in cereal plants [3]. Previous studies on the adaptive role of flavonoids in cereals have mainly been reported in barley [14, 30–35], wheat [36–40] and rice [41–43], maize [44–47]. These studies generally can be divided into two categories: the measurement of flavonoid content changes and the transcriptional responses of the flavonoid biosynthetic gene under biotic or abiotic stress conditions. Given the constant selection pressure confronting cereal plants during evolution, the evidence of environmental adaptation at the gene molecular level should theoretically also be prevalent in the flavonoid biosynthetic pathway. In the present study, we aim to explore the potential interaction between cereal plants and the environment from the gene evolutionary dynamics perspective. We focused on the F3’H gene subfamily, which encoded a critical enzyme that controls the hydroxylation patterns of the flavonoid B-ring.

Lineage-specific evolution is an important mechanism for gene evolution via duplication. It is a common observation during plant evolution and diversification. Facilitated by the latest whole genome sequencing data of barley, rye, wheat and other wheat relatives, this study presented a comprehensive genome-wide survey and a systematic phylogeny analysis on F3’H genes in 12 monocot species. We found that the distribution of putative *F3’H*s was highly unbalanced among monocots. For *Triticeae*, it ranges from 2 copies in *T. urartu* to 9 copies in *T. aestivum*. A high volatility of *F3’H* number was also observed for non-*Triticeae*, with *S. bicolor* and *B. distachyon* retaining 5 and 4 copies, respectively. In this study, we identified 3 *F3’Hs* in barley genome, in contrast to the study by Vikhorev [24], which reported only 2 copies of *F3’Hs*. In comparison, we identified an extra *F3’H* (HORVU7Hr1G095900.47) on chromosome 7H of barley, which was collinear with AET7Gv20999800 and TuG1812G0700004460, located on chromosomes Aet7 and Tu7 of *A. tauschii* and *T. urartu*, respectively. Our results are consistent with the study by Lam [25], who also identified 3 *F3’Hs* in barley. The expansion of F3’Hs in monocots was found to be mainly attributed to dispersed gene duplication. This finding is consistent with the observations made with another three gene families (MYB, MYC and F3’5’H) from the anthocyanin biosynthetic pathway in monocot plants [48]. Noteworthy, we found that tandem duplication and proximal duplication have contributed to the expansion of F3’Hs specifically in *S. bicolor* and *O. sativa*. In addition, one WGD/Segmental duplication was observed for Zm00008a016611 and Zm00008a022212 in *Z. mays*. These observations may have reflected the species-specific evolutionary history of *F3’Hs* in these three plants. Notably, despite 5 *F3’Hs* were present in *S. bicolor* genome, one *F3’H* Sobic.004G200833 may not be functional due to the loss of the critical substrate recognition sites SRS6.

Phylogeny analyses in the present study revealed that monocot F3’Hs have evolved into two independent lineages: Mono_F3’H Class I and Mono_F3’H Class II, with the previously characterised CYP75B4 in rice classified as a Class II F3’H. This finding is consistent with the report by Lam [25], which found that CYP75B4 belonged to separate phylogeny clade, and had obtained novel 5′-hydroxylase activity on chrysoeriol. Here, we showed that the divergence between Class I and Class II *F3’Hs* has originated from a duplication event predicating the species diversification of monocots. In addition, an additional duplication event could also be proposed for both Class I and Class II F3’Hs during the common ancestor of *Triticeae*, leading to the formation of 2 sub-branches in each F3’H class in *Triticeae*. The identified duplication events for Class I and Class II F3’Hs in *Triticeae* may point to a shared genome-wide duplication event in the common ancestor of *Triticeae*. Interestingly, the conservations of Class I/Class II F3’Hs and the different sub-branches in *Triticeae* both displayed unequal distribution among different species. Whilst the 2 sub-branches of Class II F3’Hs were both preserved in all *Triticeae* species included in the present study, the secondary sub-branch of Class I was found to be absent in *H. vulgare* and *A. tauschii*. This gene absence might be due to gene loss after duplication, which is a common observation in plant genomes [22, 23]. Taken together, these findings showed that F3’Hs in monocot have underwent volatile lineage-specific gene duplication and gene loss events.

As the focus of the present study, evolutionary dynamic analyses showed that monocot Class II F3’Hs had been clearly affected by positive natural selection. The detection of positive selection in Class II F3’H within this study presented direct evidence that the evolution of flavonoid biosynthetic pathway has been affected by environmental selection. F3’H and F3’5’H are close homologs in the cytochrome P450 superfamily, responsible for the production of red and blue anthocyanins, respectively. A similar observation has been made on monocot F3’5’H gene subfamily [48], in which positive selection has been shown to drive the emergence of a separate *F3’5’H* lineage responsible for the accumulation of blue anthocyanins in *Triticeae* grains. The selection on monocot *F3’5’H* was suggested to have resulted from plants’ adaptation to strong light or heat stresses. The detection of positive selection in both *F3’H* and *F3’5’H* subfamilies lend support to our earlier hypothesis that the evidence of plant-environmental interaction at the gene evolutionary level should be prevalent in the flavonoid biosynthetic pathway. Unlike monocot F3’5’H, which displayed selection for increased protein thermostability, the divergence between Class I and Class II F3’Hs seems to be more related with enzyme function at the protein structural level. This is supported by the results from the sequence alignment and protein modelling analyses in the present study. The detection of positive selection in both F3’H and F3’5’H families is corroborated by a recent report which showed that light environment may induce differences in photoprotective phenolic compounds during long-term photoacclimation [49].

F3’H is among the poorly understood flavonoid biosynthetic genes in monocot plants. Characterization of F3’H has only been reported for maize *Pr1* [50, 51], controlling the red aleurone colour; for rice CYP75B3 and CYP75B4 [25, 26], underlying the 3′-hydroxylated flavonoids and tricin formation; and for sorghum [52–54], involved in 3-deoxyanthocyanidins biosynthesis. Interestingly, CYP75B3 and CYP75B4 in rice, classified as Class I and Class II, respectively, have been shown to have divergent enzyme activity [25, 26]. In particular, the Class II F3’H CYP75B4 was shown to display novel chrysoeriol-specific 5′-hydroxylase activity and played an indispensable role in tricin biosynthesis [25]. The recruitment of 5′-hydroxylase activity for CYP75B4 was suggested to have contributed to the prevalence of tricin-derived metabolites in grasses and monocots, which have important function in plant-defence mechanisms. These observations suggested that CYP75B4 may obtained a novel biological role due to protein functional divergence. Indeed, Class II F3’Hs in monocots including CYP75B4 were found to be affected by positive natural selection, acting 7 specific amino acid sites. Protein modelling and amino acid property analyses showed that majority of these selected sites were located at the catalytic cavity of F3’H, and may have a direct effect on substrate specificity. These findings may provide an evolutionary and protein structural explanation to the observed enzyme activity differences between CYP75B3 and CYP75B4 [25, 26]. Notably, the molecular basis underlying the functional difference between F3’H and F3’5’H has been well-characterised [27]. Amino acid substitutions at two critical sites in SRS6 were shown to control the 3′- and 5′- hydroxylase activities. Interestingly, sequence alignment in our study showed that Class I and Class II F3’Hs displayed distinct amino acid substitutions at these two sites. Intriguingly, Class II F3’Hs contained a Phe at position 446, which was commonly observed for F3’5’Hs. In contrast, Class I F3’Hs retained Tyr at this position, consistent with previously characterised F3’Hs. These results are consistent with the observed 5′-hydroxylase activity for CYP75B4, a Class II F3’H. Instead, no 5′-hydroxylase activity has been observed for CYP75B3 (Class I F3’H) [25]. Our analyses provided a protein structural explanation for the 5′-hydroxylase activity in CYP75B4. The results presented here resembled a similar observation made in *Asteraceae*, in which some CYP75B proteins were identified to be clustered together with F3’Hs but displayed F3’5’H activities [55]. Our study indicated that the whole monocot Class II F3’Hs may have obtained the novel chrysoeriol-specific 5′-hydroxylase function.

In addition to the divergence at the protein structural level, functional divergence after gene duplication could also occur at the transcriptional level. In fact, gene expression analyses have been widely used to study many flavonoid biosynthetic genes in regard to their responses to various biotic and abiotic stressors. Among these studies, three differentially expressed F3’Hs (Class II) in sorghum leave under cutting stress [53, 54], two tissue-specific *F3’Hs* (Class I and Class II) in barley [24] and two F3’Hs (Class I and Class II) in rice [25] have been reported. Interestingly, in all cases, Class I and Class II displayed divergent expression profiles. This observation is consistent with the results from our transcriptional analyses, which also covered the three F3’H paralogs from maize. By combining these data together, although a common expression pattern for Class I and Class II *F3’Hs* across these monocot species can not be drawn at the moment, a transcriptional divergence for the two *F3’H* lineages may be proposed. The observation of gene functional divergence at both protein structural and gene transcriptional level have been reported in many plant gene families [56–58], and consolidated the theory of gene evolution via duplication [22, 23]. In this study, the expression of different F3’H paralogs may have evolved a species-specific profile, meeting the particular environmental challenges faced by different plants. For example, we found one of the Class I *F3’Hs* identified from sorghum displayed a clear transcriptional response to pathogen infection but was not expressed at all under normal growing condition. This result is corroborated by previous studies on *F3’Hs* in sorghum [52–54]. In addition to monocot plants, transcriptional divergence has also been reported for paralogous F3’Hs and F3’5’Hs in several eudicot plants, such as F3’Hs in tea tree leave [59], which further confirmed that functional divergence at the gene transcriptional level is a common observation during gene evolution via duplication. An evolutionary explanation for this observation would be that, the development of the complex expression profile for flavonoid biosynthetic genes resulted from the environmental selection pressure acting on different plants, and also have in turn improved plants’ survivability in nature.

## Conclusions

Based on the results from the genome-wide survey, phylogeny, evolutionary dynamics and protein structural modelling analyses, we found that monocot *F3’Hs* had underwent volatile lineage-specific gene duplication and gene loss events in monocot plants. We concluded that monocot F3’Hs have evolved into two independent lineages (Mono_F3’H Class I and Class II), after gene duplication during the common ancestor of monocot plants. The functional divergence of monocot F3’H Class II has been affected by positive natural selection, acting on several specific amino acid sites. The amino acid substitutions at these selected sites and other sites in SRS6 displayed high potential to affect the substrate binding of F3’Hs, and may have contributed to the recruitment of chrysoeriol-specific 5′-hydroxylation activity in F3’H Class I, as evidenced by CYP75B4 in rice. In addition, transcriptional divergence between F3’H Class I and Class II have also been observed. Taken together, our study revealed clear evidence of plant-environmental interaction for the flavonoid biosynthetic pathway at the gene evolutionary level.

## Methods

### Sequence retrieval and genuine F3’H homolog identification

Due to the close homology between F3’5’H and F3’H, genuine F3’H homologs were identified by a method as described in a previous study [48]. Twelve monocot crops and twelve eudicot species were included. The amino acid sequence of the previously characterised CYP75B3 homolog in barley was used as queries for BLASTP (*E*-value threshold: 1e-30) against public databases of monocot plant genomes. Remote F3’H homologs were also retrieved from lower plant moss (*P. pattens*). The overall process is as following: all homologs identified above were included for the development of a preliminary Neighbour Joining tree using MEGA7.0 software [60]. Sequence alignment was performed using Muscle [61]. The substitution model used is P-distance. 1000 times bootstrapping iteration was performed for tree assessment. The distinct phylogeny branch containing the previously reported F3’Hs (CYP75B3 & CYP75B4) in rice was selected as the genuine F3’Hs and was used for further analyses.

### Phylogeny reconstruction

The CDS sequences of the above identified F3’Hs and the remote F3’H homologs in patten were used for phylogeny reconstruction. Codon-based sequence alignment of the CDS sequences was performed using Muscle with 8 iterations [61]. The resulted sequence alignment was checked manually to remove significant alignment gaps and 5′ signal peptides. The phylogeny was searched by Bayesian simulations implemented in BEAST2 [62] under strict molecular clock assumption. The unlinked substitution model Yule + G (5 categories) was used. A single Markov Chain - Monte Carlo Chain was run for 1000,000 generations. Trees were sampled every 100 generations with 1, 000 pre burn-in until convergence. The final phylogenetic tree was inferred by TreeAnnotator with the first 1000 trees discarded. All phylogenetic trees in the present study were annotated using FigTree (http://tree.bio.ed.ac.uk/software/figtree/).

### Gene structural analyses

The CDS and genomic sequences for *F3’Hs* were downloaded from the corresponding genome database for the target monocot species. The gene structural diagram was constructed using the GSDS v2.0 online tool (http://gsds.cbi.pku.edu.cn/). The phylogenetic subtree for monocot F3’Hs developed in the present study was also used as an input to cluster the gene structures based on phylogeny relationship.

### Gene duplication pattern and colinearity analyses

The MCScanX package [63] was used to characterise the gene duplication pattern. The original genomic data was downloaded from public database and was further processed to generate the input files for MCScanX. Intra- and inter-species genome comparisons were performed using the standalone NCBI-BLAST-2.2.29 tool with an *E*-value threshold of 1e-05. Intra-genome all-vs-all BLAST was performed for gene duplication pattern identification. For colinear gene pair identification, genome dataset from different species were combined for all-vs-all BLAST.

### Sequence alignment and protein modelling

Amino acid sequence alignment and annotation were carried out using ESPript 3.0 (http://espript.ibcp.fr/ESPript/ESPript/index.php) Homologous structure template was identified by BLASTp against the PDB database using the amino acid sequences of rice F3’Hs as queries. The protein structure of CYP76AH1 (PDB: 5YM3) with the highest amino acid identity was used for the model development. The protein models of F3’Hs were created by homology modelling using the Modeller server. 5 structural models were generated for each protein. The best model was selected based on the lowest Discrete Optimized Protein Energy (DOPE) values and GA 341 score of 1, which indicate reliability of these models. The final model was validated by Ramachandran plot analysis using PROCHECK (http://www.ebi.ac.uk/thornton-srv/software/PROCHECK). Molecular visualizations were performed using PyMOL (Version 1.3r1. Schrodinger, LLC).

### Transcriptional data mining

The transcriptional data for the target *F3’H* genes was extracted from individual databases: for rice (http://expression.ic4r.org/index), barley (https://apex.ipk-gatersleben.de/apex/f?p=284:10), maize (https://www.maizegdb.org/) and sorghum (http://sorghum.riken.jp/morokoshi/Home.html).

## Additional file

Additional file 1: Amino acid sequence alignment of plant F3’Hs. (PDF 50 kb)

## Data Availability

The datasets generated and/or analysed during the current study are available in the Figshare repository at https://figshare.com/s/a811638eb6fcd3496d2f.

## Abbreviations

CDS: Coding domain sequence
CHI: Chalcone isomerase
CHS: Chalcone synthase
DAP: Days after pollination
DOPE: Discrete Optimized Protein Energy
F3’H: Flavonoid 3′-hydroxylase
F3′5’H: Flavonoid 3’5’-hydroxylase
LRTs: Likelihood Ratio Tests
SRS: Substrate recognition sites
WGD: Whole genome duplication
