# Supplementary material for: Evolutionary dynamic analyses on monocot flavonoid 3′-hydroxylase gene family reveal evidence of plant-environment interaction
Source: BMC Plant Biol. 2019 Aug 8;19:347. doi: 10.1186/s12870-019-1947-z (PMC6686259; doi:10.1186/s12870-019-1947-z)
Supplement: Supplementary file 1 — Amino acid sequence alignment of plant F3’Hs. (PDF 50 kb) [file 12870_2019_1947_MOESM1_ESM.pdf]

Supplementary file 1. Amino acid sequence alignment of plant F3'Hs. Amino acid site is numbered according to HORVU6Hr1G002400.1 without the first 35 amino acids. The positively selected sites were indicated by solid black triangle. Amino acid sites within 5 Å of the substrates were indicated by the blue star symbol. Substrate recognization site (SRS) was boxed in black line rectangle. Two amino acid residues known to reverse the enzyme activity between F3'H and F3'5'H were highlighted in yellow.

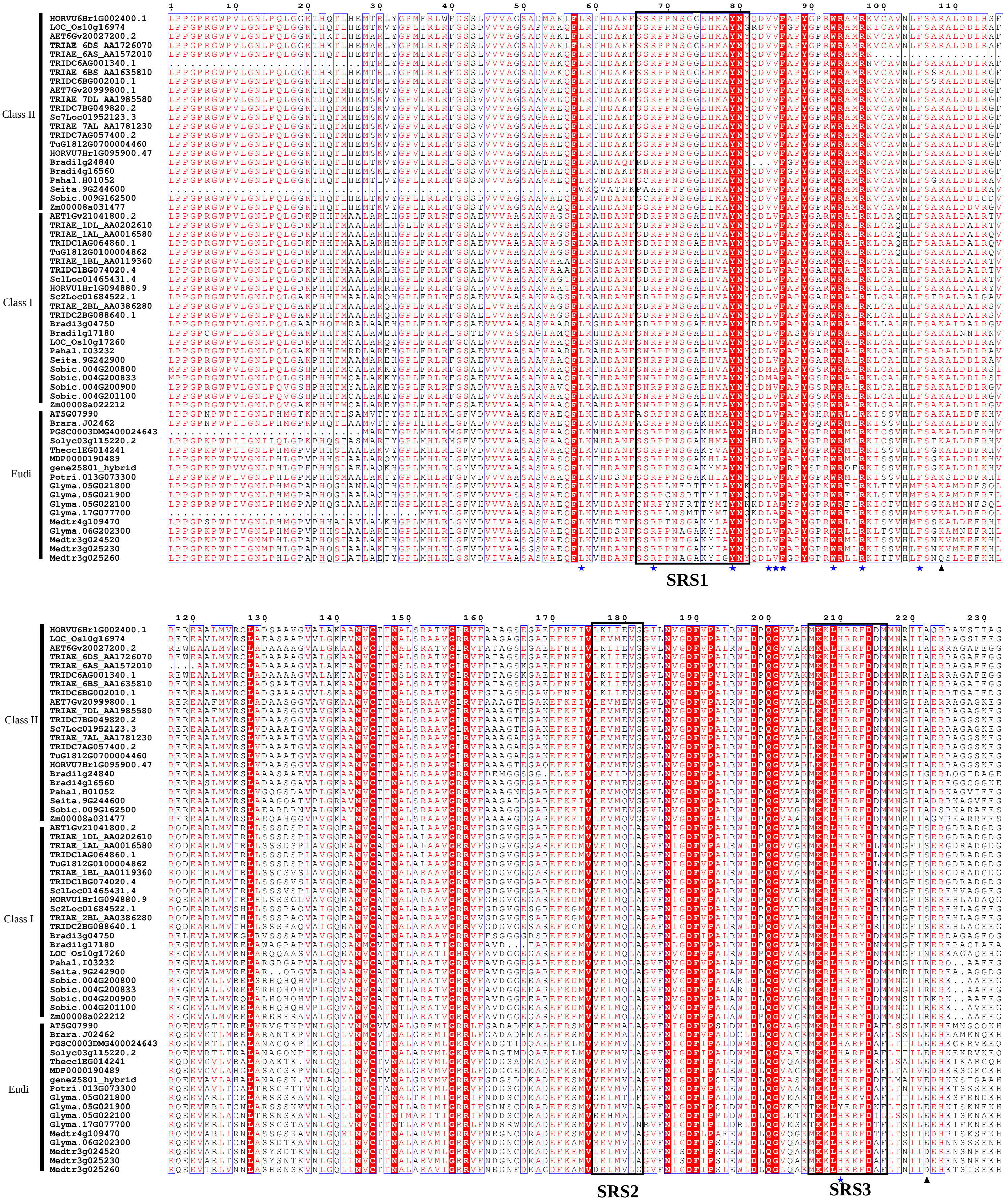

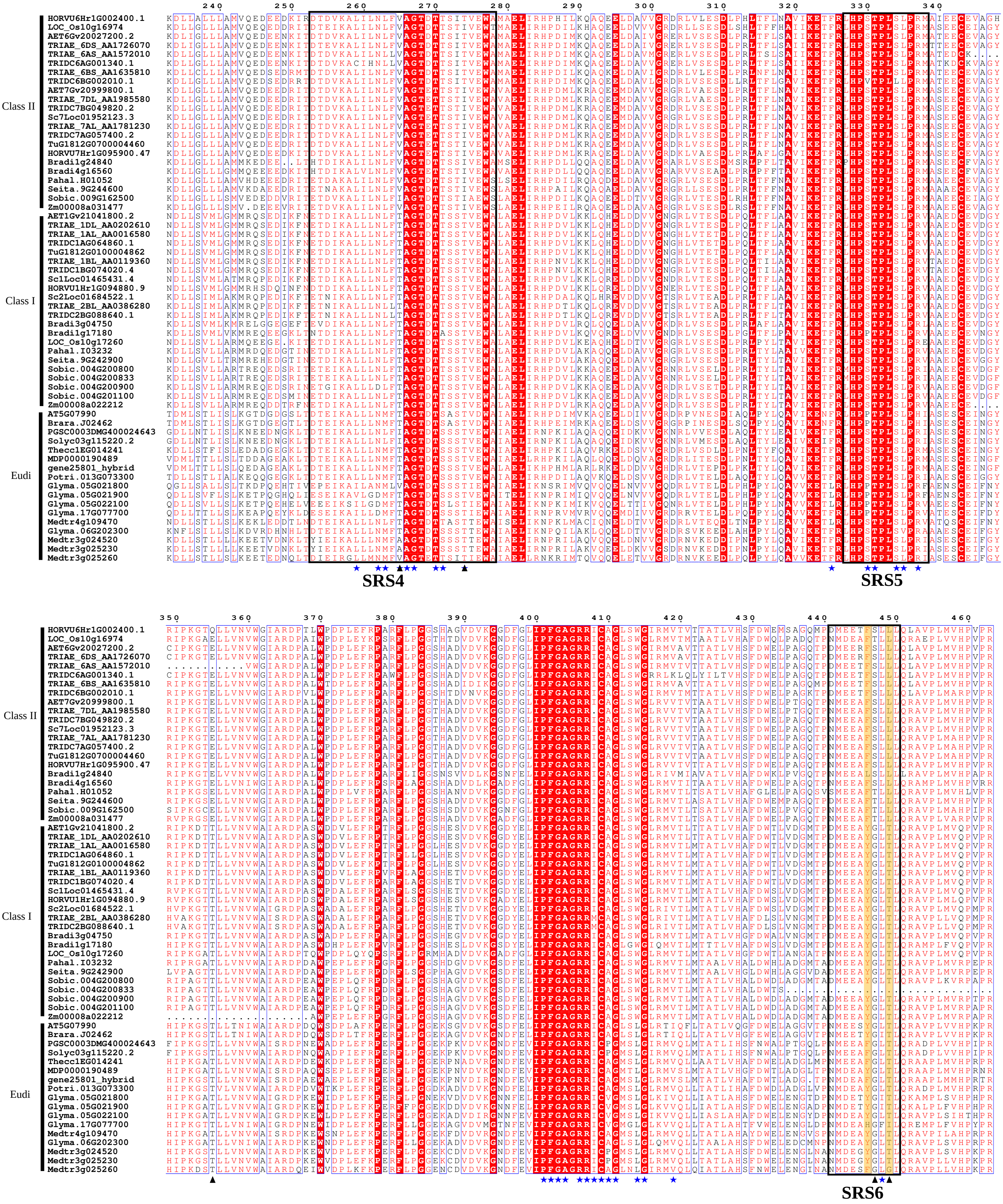
Class II
Class I
Eudi
SRS4
SRS5
SRS6
